# Correction to: Key summary of German national treatment guidance for hospitalized COVID‑19 patients: Key pharmacologic recommendations from a national German living guideline using an Evidence to Decision Framework (last updated 17.05.2021)

**DOI:** 10.1007/s15010-021-01665-y

**Published:** 2021-08-19

**Authors:** Jakob J. Malin, Christoph D. Spinner, Uwe Janssens, Tobias Welte, Steffen Weber-Carstens, Gereon Schälte, Petra Gastmeier, Florian Langer, Martin Wepler, Michael Westhoff, Michael Pfeifer, Klaus F. Rabe, Florian Hoffmann, Bernd W. Böttiger, Julia Weinmann-Menke, Alexander Kersten, Peter Berlit, Marcin Krawczyk, Wiebke Nehls, Falk Fichtner, Sven Laudi, Miriam Stegemann, Nicole Skoetz, Monika Nothacker, Gernot Marx, Christian Karagiannidis, Stefan Kluge

**Affiliations:** 1 Division of Infectious Diseases, Department I of Internal Medicine, University of Cologne, Cologne, Germany; 2 Department of Internal Medicine II, School of Medicine, Technical University of Munich, University Hospital Rechts Der Isar, Munich, Germany; 3 Medical Clinic and Medical Intensive Care Medicine, St Antonius Hospital, Eschweiler, Germany; 4 Department of Respiratory Medicine and German Centre of Lung Research (DZL), Hannover Medical School, Hannover, Germany; 5 Department of Anesthesiology and Operative Intensive Care Medicine (CCM/CVK), Charité-Universitätsmedizin Berlin, Berlin, Germany; 6 Department of Anaesthesiology, Medical Faculty Aachen, Rhenish Westphalian Technical University (RWTH), Aachen, Germany; 7 Institute of Hygiene and Environmental Medicine, Charité-University Medicine, Berlin, Germany; 8 Department of Hematology and Oncology, University Medical Center Hamburg-Eppendorf, Hamburg, Germany; 9 Department of Anesthesiology and Intensive Care Medicine, University Hospital Ulm, Ulm, Germany; 10 Department of Pneumology, Intensive Care and Sleep Medicine, Hemer Lung Clinic Centre of Pneumology and Thoracic Surgery, 58675 Hemer, Germany; 11 Department of Internal Medicine II, University Hospital Regensburg, Regensburg, Germany; 12 Department of Pneumology, Donaustauf Hospital, Donaustauf, Germany; 13 LungenClinic Grosshansdorf, Airway Research Centre North, German Centre for Lung Research, Grosshansdorf, Germany; 14 Department of Pediatrics, Dr. von Hauner Children’s Hospital, University Hospital, Ludwig-Maximilians-University Munich, Munich, Germany; 15 Department of Anesthesiology and Intensive Care Medicine, Medical Faculty and University Hospital Cologne, University of Cologne, Cologne, Germany; 16 Division of Nephrology, Department of Internal Medicine I, University Medical Center of the Johannes Gutenberg University, Mainz, Germany; 17 Department of Cardiology, Angiology and Intensive Care, Medical Faculty RWTH Aachen, Aachen, Germany; 18 Germany German Society of Neurology, Berlin, Germany; 19 Department of Medicine II, Saarland University Medical Center, Saarland University, Homburg, Germany; 20 Department of Palliative Care and Geriatric Medicine, Helios Clinic Emil Von Behring, Berlin, Germany; 21 Department of Anesthesiology and Intensive Care, University Hospital of Leipzig, Leipzig, Germany; 22 Department of Infectious Diseases and Respiratory Medicine, Charité-Universitätsmedizin Berlin, Berlin, Germany; 23 Evidence-Based Oncology, Department I of Internal Medicine and Center for Integrated Oncology Aachen Bonn Cologne Dusseldorf, Faculty of Medicine, University Hospital Cologne, University of Cologne, Cologne, Germany; 24 Institute for Medical Knowledge Management, Association of the Scientific Medical Societies (AWMF), Universität Marburg, Marburg, Germany; 25 Department of Intensive Care Medicine and Intermediate Care, Medical Faculty, University Hospital RWTH Aachen, Aachen, Germany; 26 Department of Pneumology and Critical Care Medicine, Cologne-Merheim Hospital, ARDS and ECMO Centre, Kliniken Der Stadt Köln, Witten/Herdecke University Hospital, Cologne, Germany; 27 Department of Intensive Care, University Medical Center Hamburg-Eppendorf, Hamburg, Germany

## Correction to: Infection 10.1007/s15010-021-01645-2

The original version of this article, published on July 6, 2021, contained a mistakes.

The affiliation of Dr. Laudi was assigned incorrectly. The correct information is given below.

The title and subtitle of the article were displayed incorrectly. The correct information is given above.

The original article has been corrected.

